# Comparison of early warning scoring systems for predicting stroke occurrence among hospitalized patients: A study using smart clinical data warehouse

**DOI:** 10.1371/journal.pone.0316068

**Published:** 2025-01-08

**Authors:** Chulho Kim, Jae Jun Lee, Jong-Hee Sohn, Jong-Ho Kim, Dong-Ok Won, Sang-Hwa Lee

**Affiliations:** 1 Department of Neurology, Chuncheon Sacred Heart Hospital, Hallym University College of Medicine, Chuncheon, South Korea; 2 Institute of New Frontier research Team, Hallym University, Chuncheon, South Korea; 3 Department of Anesthesiology and Pain Medicine, Chuncheon Sacred Heart Hospital, Hallym University College of Medicine, Chuncheon, South Korea; 4 Deparment of Artificial Intelligence Convergence, Hallym University, Chuncheon, South Korea; University of Pennsylvania Perelman School of Medicine, UNITED STATES OF AMERICA

## Abstract

**Background:**

This study aimed to evaluate the predictive ability of two widely used early warning scoring systems, the Modified Early Warning Score (MEWS) and the National Early Warning Score (NEWS), for predicting stroke occurrence in hospitalized patients.

**Methods:**

The study enrolled 5,474 patients admitted to the intensive care unit from the general ward using data from the Smart Clinical Data Warehouse (CDW). MEWS and NEWS were calculated based on vital signs and clinical parameters within four hours of stroke onset. Stroke occurrence was categorized as ischemic or hemorrhagic. Logistic regression and receiver operating characteristic curve analyses were performed to assess the predictive abilities of the scoring systems.

**Results:**

Of the enrolled patients, 33.9% (n = 1853) experienced stroke, comprising 783 cases of ischemic stroke and 1,070 cases of hemorrhagic stroke. Both the MEWS and the NEWS were found to significantly predict overall stroke occurrence with a cutoff value of 4 (MEWS>4; OR [95% CI]: 13.90 [11.51–16.79], p<0.001; NEWS>4; OR [95% CI]: 6.71 [5.75–7.83], p<0.001). Parameters, such as prior malignancy, atrial fibrillation, AVPU response, heart rate, respiratory rate, and oxygen saturation, are also associated with stroke occurrence. The predictive ability of MEWS and NEWS was good for overall stroke occurrence. (AUC of MEWS: 0.92, 95% CI [0.91–0.93], p<0.001; AUC of NEWS: 0.85, 95% CI [0.84–0.86], p<0.001). The predictive ability was considered fair for ischemic stroke but good for hemorrhagic stroke.

**Conclusion:**

MEWS and NEWS demonstrated significant predictive abilities for overall stroke occurrence among hospitalized patients, with MEWS slightly outperforming NEWS.

## Introduction

The incidence of in-hospital stroke has been reported to range from 2.2% to 17% of all strokes in hospitalized patients [1, 2]. In-hospital stroke is associated with an unfavorable prognosis, with mortality rates higher than those for community-onset stroke, and complications more frequent, limiting improvements in functional status after stroke [1, 3–6]. Additionally, large vessel occlusion and greater stroke severity occur more frequently in in-hospital stroke cases [1, 3, 4]. Therefore, early detection and intervention are critical to improving stroke prognosis in hospitalized patients. However, time delays in recognizing stroke symptoms and initiating reperfusion therapy in in-hospital stroke patients than in community-onset stroke patients [7]. This delay is attributed to poor recognition of stroke due to factors such as anesthesia, sedation, and the serious illnesses caused by comorbidities, which limit access to neurological examinations [3, 7–9]. Hence, detection modality of stroke in hospitalized patients with medical and surgical conditions that affect misdiagnosis for neurological deterioration during hospitalization is needed.

Early warning scores and systems are widely used in clinical practice by hospital rapid response teams to identify patients at high risk of deterioration [10]. Among these systems, the National Early Warning Score (NEWS) [11] and the Modified Early Warning Score (MEWS) are commonly used tools to identify patients at risk for catastrophic events, including death [12]. Most previous studies have focused on the usefulness of NEWS and MEWS in predicting in-hospital mortality, complications, and intensive care unit (ICU) admission in hospitalized patients with medical conditions [12–17]. However, few studies have evaluated the association between early warning scores and the prognosis of hospitalized stroke patients [18, 19]. Since both scoring systems include assessments of level of consciousness and various vital signs, we hypothesized that these systems could predict stroke occurrence in hospitalized patients with medical conditions.

This study presents a novel approach to evaluate the predictive ability of two early warning systems, MEWS and NEWS, for the occurrence of stroke in hospitalized patients, using a large real-world dataset from an intelligent CDW. This study also provides new insights into how these scoring systems predict different types of stroke by comparing the ischemic and hemorrhagic stroke subtypes in detail. Using real-world data, we aimed to increase the generalizability of our findings and add a unique perspective to the existing literature on stroke prediction.

## Materials and methods

### Study population

We retrospectively collected clinical data using Hallym University Medical Center’s (HUMC) clinical big data analytics platform, Smart CDW. Smart CDW, based on the QlikView Elite solution (Qlik, Lund, Sweden), has been operational in HUMC’s five hospitals since 2013. HUMC’s shared CDW system provides comprehensive electronic medical record data, including physical measurements, medication history, diagnosis, therapy, imaging data, serial vital signs, and laboratory data from over 10 years. We accessed the CDW system and collected clinical data from all hospitalized patients at HUMC-affiliated hospitals (Hallym Sacred Heart Hospital, Kangnam Sacred Heart Hospital, Dongtan Sacred Heart Hospital, Chuncheon Sacred Heart Hospital, and Hangang Sacred Heart Hospital) from January 2016 to June 2023. We enrolled patients who were transferred from general wards to the ICU. Imaging data (brain computed tomography [CT] and magnetic resonance imaging [MRI]) were extracted from these patients, and the occurrence of stroke, including ischemic and hemorrhagic stroke, was identified.

### Definition of parameters and outcome measures

HUMC’s electronic CDW system was used to obtain data on patient demographics, underlying systemic comorbidities, and physical measurements including level of consciousness, serial vital signs, and medication history. In order to organize the study design to resemble real-world practice, we enrolled patients who were transferred from the general ward to the ICU.

Demographic characteristics included patient age and sex at admission. Systemic comorbidities included hypertension, diabetes mellitus (DM), coronary artery disease, previous stroke, dyslipidemia, underlying malignancy, and atrial fibrillation (AF).

Physical measurements included levels of consciousness based on the Glasgow Coma Scale (GCS) and serial vital signs, including systolic blood pressure (SBP), diastolic blood pressure (DBP), heart rate (HR), oxygen saturation (SaO2), respiratory rate (RR), and body temperature (BT). All HUMC-affiliated hospitals had a Rapid Response Team (RRT) system that was activated based on NEWS scores. We collected NEWS that activated the RRT system within 4 h prior to transfer to the ICU and collected all items comprising the NEWS and MEWS. The MEWS consists of five physiological parameters, including SBP, HR, RR, BT, and alert, response to voice, response to pain, and unresponsive (AVPU) scores based on the GCS. The MEWS is a tool used to assess a patient’s risk of deterioration. To determine the patient’s MEWS score, each of the five vital signs was assigned a number between 0 and 3. A score greater than 4 indicated a high risk of mortality and the need for ICU transfer [12]. NEWS included the following seven vital sign parameters: RR, SaO2, oxygen supplementation, SBP, RR, BT, and AVPU score based on GCS [11]. Patients with a NEWS score greater than 4 were considered to be at medium or high risk.

The primary outcome was stroke occurrence during hospitalization, identified using brain CT and MRI at the time of transfer to the ICU. These scans were reviewed by expert vascular neurologists (S-H Lee and C Kim) and categorized them as ischemic or hemorrhagic stroke. The secondary outcome measure was the occurrence of ischemic and hemorrhagic strokes during hospitalization.

### Statistical analysis

We hypothesized that NEWS and MEWS could predict the occurrence of stroke in hospitalized patients. We present summary statistics as number of subjects (percentage) for categorical variables and as mean ± SD or median [interquartile range (IQR)] for continuous variables. The patients were divided into two groups: those who experienced a stroke and those who did not. Pearson’s chi-squared test was used for categorical variables and Student’s t-test or Mann-Whitney U test for continuous variables, as appropriate, for group comparisons.

Binary logistic regression analysis was used to assess the independent effects of NEWS and MEWS on the outcome measures. Variables for multivariate analysis were selected based on a combination of clinical relevance and statistical significance from univariate analysis. Specifically, variables with a p-value < 0.1 in the univariate analysis were considered for inclusion in the multivariate model. In addition, clinically important factors, such as age, sex, AF, malignancy, and prior stroke, were included in the model regardless of their statistical significance in the univariate analysis because of their well-established association with stroke risk. This approach ensured comprehensive representation of both statistically significant and clinically relevant predictors in the final model. Crude and adjusted odds ratios (ORs) and 95% confidence intervals (CIs) were calculated. In addition, we examined the effects of NEWS and MEWS on ischemic and hemorrhagic strokes separately using a logistic regression model.

To evaluate the predictive ability of NEWS and MEWS for stroke occurrence, we performed receiver operating characteristic (ROC) curve analysis using the ’pROC’ package in R. We calculated the 95% confidence interval (CI) for the area under the curve (AUC) and p-value using the Delong’s test. The cutoff values for NEWS and MEWS for predicting stroke occurrence were determined using the Youden index. In addition, ROC curve analyses were performed to assess the predictive ability of NEWS and MEWS for the occurrence of ischemic and hemorrhagic stroke.

For the sensitivity analysis, we performed propensity score matching (PSM) to account for potential covariate imbalance and confounding factors between patients with and without stroke. The propensity score for each group was calculated as the probability of stroke occurrence based on patients’ baseline demographics and vascular risk factors in the initial logistic regression analysis. Patients with and without stroke were matched 1:1 using the nearest-neighbor method based on these propensity scores. A binary logistic regression analysis was performed using PSM cohorts to evaluate the effects of MEWS and NEWS on stroke occurrence. Statistical analyses were performed using IBM SPSS version 21.0 software (IBM Corporation, Armonk, NY, USA) and R version 4.0.3 moonBook and MatchIt (R Core Team 2020, R Foundation for Statistical Computing, Vienna, Austria).

### Ethics declarations

The use of the CDW database and additional medical records for this study was approved by the local institutional review board (IRB) of the four centers without the need for informed consent from patients because of the study participants’ anonymity and minimal risk to patients. (Chuncheon Sacred Heart Hospital IRB no. 2023-09-001). We first received the CDW data on September 14, 2023. The data did not contain any unique personal information.

## Results

During the study period, 39,474 patients admitted to the ICU were reviewed. We excluded patients admitted to the ICU via the emergency department (n = 32,505), patients with unavailable vital signs within 4 hours of stroke onset (n = 1102), patients with unavailable brain imaging (n = 393) from the analysis. The remaining 5,474 patients were enrolled in the study.

### Overall stroke prediction

**Fig 1:**
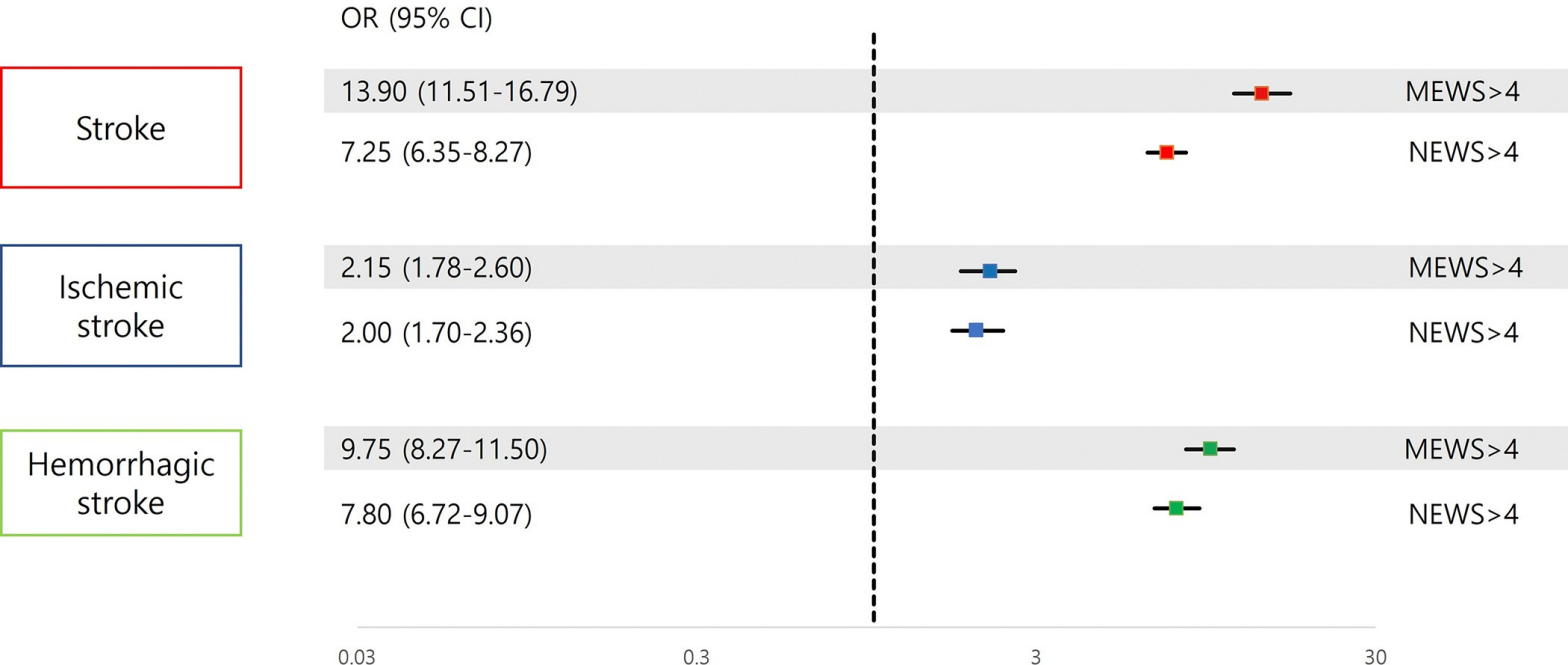
Multivariate analysis showing impact of MEWS and NEWS on stroke occurrence. Abbreviation: MEWS, Modified Early Warning Score; NEWS, National Early Warning Score, OR, odd ratio; CI, confidence interval.

Of the 5,474 enrolled patients, 33.9% (n = 1,853) experienced a stroke during hospitalization, with 783 cases of ischemic stroke and 1,070 of hemorrhagic stroke. Patients with stroke had higher MEWS and NEWS, and patients with stroke had higher scores for each parameter of MEWS and NEWS than patients without stroke (Table 1). Furthermore, the MEWS and NEWS scores of patients with hemorrhagic stroke were higher than those of patients with ischemic stroke (Table 1 and S1 Table). In multivariate analysis, both MEWS and NEWS showed strong predictive ability for overall stroke occurrence. MEWS showed slightly superior predictive performance compared to NEWS, with MEWS >4 associated with a markedly increased odds ratio for stroke occurrence (OR [95% CI]: 13.90 [11.51–16.79], p<0.001), while NEWS >4 had an odds ratio of 7.25 ([95% CI]: 6.35–8.27, p<0.001) [Fig 1, S2 and S3 Tables]. These findings highlight that while both systems are effective, MEWS offers slightly superior predictive performance.

**Table 1:** Baseline characteristics between patients with overall stroke occurrence and those without stroke occurrence.

	Total cohort			PSM cohort		
	Stroke (-) (n = 3621)	Stroke (+) (n = 1853)	p-value	Stroke (-) (n = 1853)	Stroke (+) (n = 1853)	p-value
Age, n (SD)	64.5 (15.0)	66.8 (14.9)	0.32	67.3 (14.3)	66.8 (14.9)	0.29
Male, n (%)	1916 (52.9)	998 (53.9)	0.51	959 (51.8)	998 (53.9)	0.21
HTN, n (%)	1800 (49.7)	930 (50.2)	0.75	952 (51.4)	930 (50.2)	0.49
DM, n (%)	899 (24.8)	525 (28.3)	0.01	561 (30.3)	525 (28.3)	0.21
CAD, n (%)	325 (9.0)	183 (9.9)	0.28	165 (8.9)	183 (9.9)	0.34
Prior stroke, n (%)	411 (11.4)	189 (10.2)	0.20	181 (9.8)	189 (10.2)	0.70
Prior malignancy, n (%)	474 (13.1)	356 (19.2)	<0.001	412 (22.2)	356 (19.2)	0.03
Hyperlipidemia, n (%)	384 (10.6)	176 (9.5)	0.20	176 (9.5)	176 (9.5)	1.00
AF, n (%)	123 (3.4)	252 (13.6)	<0.001	123 (6.6)	252 (13.6)	<0.001
Alcohol, n (%)	775 (21.4)	309 (16.7)	<0.001	278 (15.0)	309 (16.7)	0.18
Smoking, n (%)	598 (16.5)	234 (12.6)	<0.001	215 (11.6)	234 (12.6)	0.37
Prior antithrombotic, n (%)	512 (14.1)	263 (14.2)	0.97	248 (13.4)	263 (14.2)	0.51
MEWS, n (%)			<0.001			<0.001
0–4	3466 (95.7)	1138 (61.4)		1761 (95.0)	1138 (61.4)	
>4	155 (4.3)	715 (38.6)		92 (5.0)	715 (38.6)	
NEWS, n (%)			<0.001			<0.001
0–4	3.056 (84.4)	783 (42.3)		1525 (82.3)	783 (42.3)	
>4	565 (15.6)	1070 (57.7)		328 (17.7)	1070 (57.7)	

Abbreviation: PSM, propensity score matching; SD, standard deviation; HTN, hypertension; DM, diabetes mellitus; AF, atrial fibrillation; MEWS, modifies early Warning Score; NEWS; National Early Warning Score

**Fig 2:**
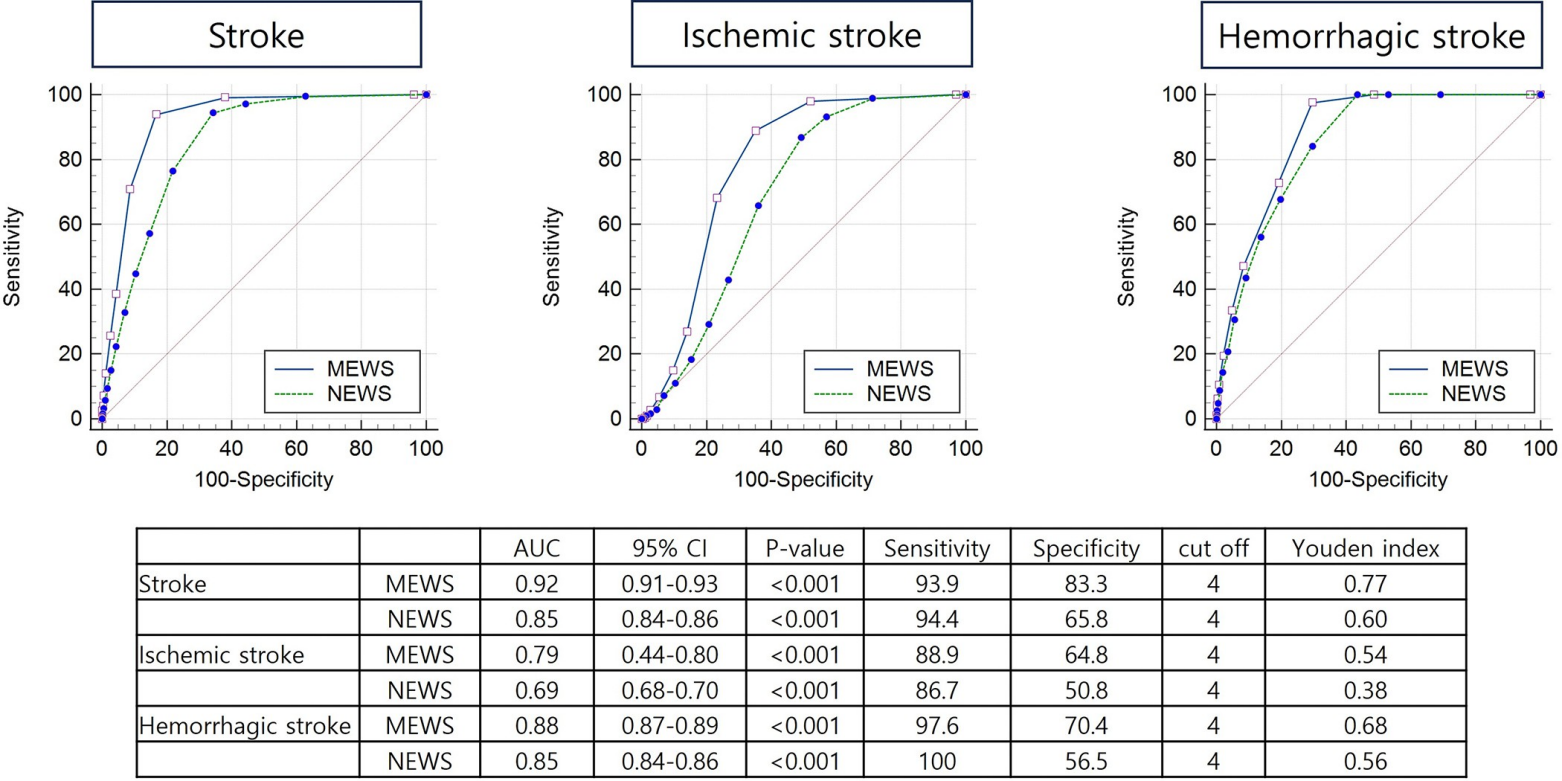
ROC curve showing the predictive ability of MEWS and NEWS for stroke occurrence. Abbreviations: ROC, receiver operating curve; MEWS, Modified Early Warning Score; NEWS, National Early Warning Score, AUC, area under curve.

The AUC analysis corroborated these findings. MEWS achieved an AUC of 0.92 (95% CI [0.91–0.93], p<0.001), while NEWS had an AUC of 0.85 (95% CI [0.84–0.86], p<0.001), indicating stroke discriminative power for overall stroke prediction (Fig 2). These results suggest that both MEWS and NEWS are valuable tools for identifying patients at risk of stroke, with MEWS demonstrating a higher predictive accuracy.

### Ischemic and hemorrhagic stroke subtypes

The predictive performance of MEWS and NEWS differed when stratified by stroke subtype. For ischemic stroke, MEWS and NEWS achieved AUCs of 0.79 and 0.69, respectively, indicating fair predictive ability. This suggests that these scoring systems may be less sensitive to the more subtle, gradual onset typical of ischemic stroke. In contrast, both MEWS and NEWS performed better in predicting hemorrhagic stroke, with AUCs of 0.88 and 0.85, respectively, indicating good predictive ability (Fig 2). The better performance for hemorrhagic stroke likely reflects the acute and severe physiological changes, such as rapid neurological deterioration, that these scoring systems are designed to detect. These findings emphasize the need for tailored predictive models to improve ischemic stroke detection while leveraging the strengths of MEWS and NEWS for hemorrhagic stroke.

### Key predictive parameters

Specific parameters within MEWS and NEWS were particularly predictive of stroke occurrence. Elevated HR, decreased oxygen saturation (SaO2), and lower AVPU scores (indicating impaired consciousness) were significant contributors (Table 2). Elevated HR was strongly associated with an increased stroke risk, probably due to cardiovascular instability, especially in patients with AF. Decreased AVPU score was critical in predicting hemorrhagic stroke, reflecting rapid clinical progression. Furthermore, comorbidities such as prior malignancy and AF were independently associated with stroke risk across both subtypes, underscoring the complex interplay of systemic neurological factors influencing stroke occurrence (Table 3). These findings highlight the importance of integrating vital sign abnormalities and comorbidities into predictive models to improve early stroke detection.

**Table 2:** Multivariate analysis showing impact of MEWS and NEWS parameters on stroke occurrence.

	OR	95%CI	p-value		OR	95%CI	p-value
Clinical parameters							
Age	0.998	0.99–1.01	0.65	Age	0.999	0.99–1.004	0.70
Male	1.15	0.94–1.41	0.17	Male	1.24	1.07–1.45	0.01
DM	1.03	0.83–1.28	0.80	DM	0.96	0.81–1.13	0.61
prior malignancy	1.44	1.11–1.97	0.01	prior malignancy	1.37	1.12–1.66	0.002
AF	5.42	3.83–7.68	<0.001	AF	4.81	3.66–6.32	<0.001
Alcohol	0.97	0.73–1.29	0.83	Alcohol	0.94	0.76–1.17	0.59
Smoking	0.99	0.73–1.34	0.93	Smoking	0.91	0.72–1.16	0.46
Warning Score parameters							
MEWS parameter				NEWS parameter			
SBP	0.99	0.94–1.18	0.95	SBP	0.99	0.92–1.07	0.84
HR	5.46	4.70–6.35	<0.001	HR	2.90	2.59–3.24	<0.001
RR	1.13	0.97–1.33	0.13	RR	1.12	1.04–1.22	0.004
BT	0.87	0.69–1.09	0.23	BT	1.03	0.91–1.16	0.66
AVPU	7.3	6.58–8.09	<0.001	APVU	2.32	2.20–2.44	<0.001
				SaO2	1.39	1.26–1.55	<0.001
				O2 supplement	0.68	0.33–1.42	0.31

Abbreviation: MEWS, modifies early Warning Score; NEWS; National Early Warning Score; OR, odds ratio; CI, confidence interval; DM, diabetes mellitus; AF, atrial fibrillation; SBP, systolic blood pressure; HR, heart rate; RR, respiratory rate; BT, body temperature; SaO2, oxygen saturation; AVPU, alert, responds to Voice, responds to Pain, Unresponsive

**Table 3:** Multivariate analysis showing impact of MEWS and NEWS parameters on ischemic and hemorrhagic stroke occurrence.

	Ischemic stroke		Hemorrhagic stroke			Ischemic stroke		Hemorrhagic stroke	
	OR	95%CI	OR	95%CI		OR	95%CI	OR	95%CI
Age	0.995	0.99–1.004	1.00	0.99–1.01	Age	0.995	0.99–1.002	1.004	0.997–1.01
Male	1.08	0.85–1.37	1.1	0.82–1.47	Male	1.13	0.93–1.37	1.34	1.10–1.62
HTN	0.97	0.76–1.25	1.11	0.82–1.51	HTN	0.99	0.81–1.22	0.99	0.81–1.22
DM	1.02	0.78–1.34	1.04	0.76–1.43	DM	0.98	0.79–1.22	1.01	0.82–1.25
Prior malignancy	1.48	1.10–2.01	1.26	0.86–1.84	Prior malignancy	1.47	1.15–1.87	1.34	1.4–1.72
AF	7.77	5.51–10.96	1.02	0.52–1.99	AF	8.34	6.36–10.93	1.18	0.76–1.84
alcohol	1.08	0.78–1.49	0.79	0.50–1.25	alcohol	1.04	0.80–1.35	0.89	0.67–1.19
Smoking	0.97	0.68–1.38	0.91	0.57–1.46	Smoking	0.91	0.68–1.21	0.80	0.59–1.09
Warning score parameters									
MEWS parameters					NEWS parameters				
SBP	0.95	0.77–1.17	0.998	0.79–1.27	SBP	0.98	0.88–1.08	0.995	0.91–1.09
HR	6.60	5.59–7.79	1.33	1.11–1.59	HR	2.92	2.57–3.32	1.48	1.31–1.67
RR	0.89	0.74–1.08	1.26	1.01–1.56	RR	1.02	0.92–1.13	1.14	1.04–1.25
BT	0.92	0.72–1.19	0.89	0.64–1.24	BT	1.22	1.06–1.40	0.93	0.80–1.07
AVPU	4.27	3.85–4.74	30.25	23.66–38.68	APVU	1.51	1.42–1.61	5.89	4.81–7.19
					SaO2	1.48	1.31–1.68	1.33	1.18–1.49
					O2 supplement	0.57	0.21–1.57	0.85	0.38–1.93

Abbreviation: MEWS, modifies early Warning Score; NEWS; National Early Warning Score; OR, odds ratio; CI, confidence interval; HTN, hypertension; DM, diabetes mellitus; AF, atrial fibrillation; SBP, systolic blood pressure; HR, heart rate; RR, respiratory rate; BT, body temperature; SaO2, oxygen saturation; AVPU, alert, responds to Voice, responds to Pain, Unresponsive

### Sensitivity analysis

PSM analysis confirmed the robustness of these findings. Both MEWS >4 and NEWS >4 remained significantly associated with incident stroke occurrence in the PSM cohort (S4 Table). This supports the reliability of these scoring systems in identifying patients at risk, even after adjustment for potential confounders. The consistency of results across sensitivity analyses reinforces the utility of MEWS and NEWS as effective early warning systems for stroke prediction in hospitalized patients.

## Discussion

Our study focused on evaluating evaluated the predictive ability of two widely used early warning scoring systems, the NEWS and MEWS, to identify hospitalized patients at risk for stroke. These scoring systems, designed to identify patients at high risk of deterioration, have been widely used in clinical practice, particularly in hospital rapid-response programs. We found that both the MEWS and NEWS scores, with a cut-off of 4, significantly predicted the occurrence of stroke within four hours of onset. Given their strong predictive power, MEWS and NEWS can be used to improve decision-making in general wards and ICUs. For example, identification of patients with high MEWS or NEWS scores (4 or higher) could serve as an early signal to activate a rapid response team, which could expedite neurological assessment and facilitate diagnostic interventions such as brain imaging. This rapid recognition is particularly important to improve clinical outcomes by reducing the time to reperfusion therapy or other stroke treatments. In addition, these scoring systems can help with resource allocation by identifying patients at high risk of stroke who need closer monitoring or transfer to a higher level of care, such as an ICU. Incorporating MEWS and NEWS into hospital protocols can be in line with the current stroke management guidelines, which emphasize the importance of early detection and timely intervention to reduce stroke-related morbidity and mortality. In this context, the MEWS and NEWS serve as useful tools to guide clinical action, optimize patient management, and ultimately improve stroke care in real-world settings.

Our study found that high MEWS and NEWS scores (>4 points) increased the likelihood of stroke detection. A multivariate analysis using PSM cohorts strengthened the robustness of our findings. In addition, specific parameters within MEWS and NEWS were significantly associated with stroke occurrence. Prior malignancy, AF, and AVPU response were identified as important indicators in both scoring systems. Additionally, HR in MEWS, RR, SaO2, and HR in NEWS were predictive of stroke occurrence. When analyzing stroke subtypes, both ischemic and hemorrhagic stroke were associated with higher MEWS and NEWS scores. Changes in physiological stress may influence the risk of stroke. An elevated HR may indicate activation of the sympathetic nervous system and increased adrenergic activity, both of which can contribute to the development of stroke [20, 21]. High HR is associated with increased cardiac output, which can potentially lead to hemodynamic instability and thrombus formation [22, 23]. Elevated HR may indicate underlying cardiovascular risk factors, such as hypertension and AF, which are known to contribute to stroke occurrence [20]. Similarly, changes in RR may reflect alterations in oxygenation levels and pulmonary function, in which hypoxia is associated with respiratory distress, contributing to endothelial dysfunction and the formation of prothrombotic conditions [24]. These parameters, along with SaO2 and the AVPU scale, reflect the systemic nature of stroke, emphasizing the importance of holistic assessment in early warning scoring systems, such as MEWS and NEWS. In the future, it will be essential to advance beyond existing early warning systems and develop a scoring system that accurately predicts stroke by holistically analyzing the patient’s vital signs and underlying diseases. Notably, our findings did not negate the overall importance of SBP in determining stroke risk. Although elevated SBP at admission is common in patients with acute stroke [25], our study suggests that SBP within 4 hours before stroke alone may not be a reliable indicator of these two stroke subtypes. Future research should investigate the dynamics of blood pressure fluctuations during the prestroke and acute stroke phases, considering the temporal aspects of SBP changes in relation to stroke onset. Furthermore, the inclusion of additional hemodynamic parameters or advanced monitoring techniques may provide a more nuanced understanding of the role of blood pressure in predicting specific stroke subtypes. Elucidating the mechanisms by which these parameters contribute to stroke pathogenesis will refine early warning systems, allowing targeted interventions and improved clinical monitoring of at-risk individuals, particularly those with comorbidities such as AF and malignancy.

Several risk-scoring systems have been used in clinical practice to identify patients at risk of deterioration, including those at risk of catastrophic events such as stroke. However, two scoring systems, NEWS and MEWS, have proven to be particularly valuable tools that are widely used in hospital rapid response programs [26–28]. The advantages of NEWS and MEWS lie in their comprehensive assessment of patients, which includes multiple parameters such as vital signs and level of consciousness. These scoring systems offer a comprehensive approach to patient assessment and provide a more nuanced understanding of the risk of deterioration. Both scores include parameters such as the AVPU response, which allows for the assessment of neurological status and is critical in the context of stroke prediction. In addition, the simplicity and ease of use of NEWS and MEWS make them practical for routine clinical use [18, 29]. They provide a standardized and easy-to-use method for assessing risk, enabling healthcare providers to quickly identify patients at high risk for adverse events. This simplicity is particularly beneficial in busy clinical environments, where rapid decision-making is essential. The widespread adoption of NEWS and MEWS in various healthcare settings has generated a wealth of data that facilitates benchmarking and comparison across institutions. This improvement is due to the clinical utility of these scoring systems, which allow for continuous improvement and refinement based on real-world data and experience. Integrating these scoring systems into rapid hospital response programs can significantly improve the early identification of at-risk patients, facilitate timely interventions, and ultimately improve patient outcomes.

Receiver operating characteristic (ROC) curve analysis showed good predictive ability of both MEWS and NEWS for total stroke occurrence. The AUC values indicate excellent discriminative power, with MEWS performing slightly better than NEWS. Notably, both scoring systems showed fair predictability of ischemic stroke, whereas their performance in predicting hemorrhagic stroke was considered good. The observed discrepancy in the predictive ability of ischemic and hemorrhagic stroke may be due to the different pathophysiological characteristics of these subtypes. Ischemic stroke, commonly caused by thrombotic or embolic occlusion of the cerebral artery, is often associated with a gradual onset of symptoms and may present with subtle or nonspecific clinical signs. In contrast, hemorrhagic stroke caused by bleeding within the brain tends to present with more abrupt and severe symptoms [30]. Several factors contribute to the challenge of predicting ischemic stroke by using early warning scores. First, ischemic stroke may evolve over time, providing a window for clinical intervention before the full spectrum of symptoms becomes apparent. This gradual progression may not result in immediate and significant changes in the vital signs captured by the scoring systems. Second, ischemic stroke may occur without overt hemodynamic instability, particularly in cases where the thrombotic process is localized or limited in its impact on cerebral perfusion [31]. Conversely, the more acute and potentially catastrophic nature of hemorrhagic stroke, characterized by the sudden release of blood into the brain parenchyma, may result in more pronounced physiological derangements. The immediate impact on intracranial pressure, cerebral blood flow, and overall hemodynamics may result in more pronounced changes in vital signs, making early warning scores such as the MEWS and NEWS more effective in predicting hemorrhagic stroke. Furthermore, these differences in pathophysiology between ischemic and hemorrhagic strokes may explain the different effects of vital signs (especially HR) and sudden changes in consciousness on the two stroke subtypes in this study. An elevated HR is significantly associated with ischemic stroke due to thrombus formation and hemodynamic instability through the mechanisms mentioned above, whereas AVPU, indicating rapid neurological deterioration, is more significantly associated with hemorrhagic stroke for the same reasons. These findings highlight the need for future research to develop subtype-specific prediction models that consider the different pathophysiological mechanisms of ischemic and hemorrhagic strokes. Such models would allow for more refined risk stratification and targeted clinical interventions. Future studies could further improve the predictive power of early warning systems, such as MEWS and NEWS, by incorporating additional hemodynamic parameters and exploring blood pressure variability. These approaches will contribute to personalized and effective stroke management in the clinical setting.

Smart CDW enables the collection and retrieval of significant amounts of clinical data from multiple sources, covering a wide range of patient characteristics and medical histories. This comprehensive dataset facilitated a more in-depth analysis of the correlation between Early Warning Scores and stroke incidence, thereby improving the generalizability and practicality of the findings. In addition, the use of CDW reduces the risk of transcription errors and improves data accuracy. This digital infrastructure ensured reliable data, which strengthened the integrity of the results. Our study provides valuable insights into the effectiveness of early warning scoring systems for predicting stroke in hospitalized patients using CDW data. The strong performance of MEWS and NEWS suggests their potential utility in identifying patients at risk for stroke, allowing for timely interventions to improve outcomes. Further research and prospective validation are required to refine and optimize these scoring systems and increase their sensitivity and specificity in different clinical settings.

Although our study provides valuable insights, it is important to acknowledge some of its limitations. First, its retrospective design is a major limitation. Reliance on historical data from the Smart CDW introduces the potential for selection bias and limits our ability to establish causality. Prospective studies would provide a more robust basis for validating the predictive models developed in this study and would capture real-time data for more nuanced analyses. Second, the generalizability of the study may be affected by the specific characteristics of the patient population in the CDW dataset. Variations in clinical practice, patient demographics, and healthcare infrastructures across settings may have affected the external validity of the results. However, five of the included hospitals were affiliated with the same foundation and shared the same imaging, laboratory protocols, and other infrastructures, which allowed for maximum generalizability. In addition, we excluded patients who were transferred directly from the emergency department to the ICU to ensure that the data reflected real-world clinical practice. Third, the exclusion criteria used in our study, such as the requirement for vital signs within four hours of stroke onset and the availability of brain imaging, may have introduced selection bias. Patients with incomplete data or those excluded owing to specific criteria may differ systematically from the included population, potentially affecting the generalizability of our results. In addition, the exclusion of patients admitted to the emergency department may limit the representation of stroke cases with an acute onset. Fourth, while this study accounted for important confounders such as AF, malignancy, and prior stroke, other factors such as medication use, detailed neurological assessment, and additional comorbidities may also have affected the predictive accuracy of MEWS and NEWS. Medications, such as anticoagulants, antiplatelet agents, and sedatives, can alter vital signs and influence stroke risk, particularly in hospitalized patients. More comprehensive neurological assessments beyond the AVPU scale may also better capture the early signs of stroke-related deterioration. Future studies should incorporate these variables into more advanced prediction models to improve stroke risk stratification. Detailed medication histories, broader neurological assessments, and a wider range of comorbidities could provide a more nuanced understanding of stroke risk. This would help refine early warning systems, such as MEWS and NEWS, and ensure their effectiveness in different patient populations and clinical settings. Finally, our analysis categorized stroke into ischemic and hemorrhagic subtypes, providing a broad classification of events. However, the study did not examine the different etiologies of these subtypes or consider a more detailed classification. To gain more nuanced insights into the predictive ability of early warning scores, it is important to explore the specific characteristics of ischemic strokes, such as large vessel occlusion or cardioembolic origin, and to differentiate between the subtypes of hemorrhagic strokes.

In conclusion, this study provides valuable insights into the predictive ability of MEWS and NEWS for stroke occurrence in hospitalized patients. These findings may contribute to the evolving landscape of stroke prediction in hospitalized patients and highlight the potential role of warning scores in improving patient outcomes.

## Supporting information

S1 Table Baseline characteristics between overall stroke, ischemic and hemorrhagic stroke. (DOCX)

S2 Table Multivariate analysis showing MEWS and NEWS >4 on stroke occurrence. (DOCX)

S3 Table Multivariate analysis showing MEWS and NEWS >4 on ischemic and hemorrhagic stroke occurrence. (DOCX)

S4 Table Multivariate analysis showing MEWS and NEWS on stroke occurrence using PSM cohort. (DOCX)

S1 Dataset (XLSX)
