# Supplementary material for: Comparison of early warning scoring systems for predicting stroke occurrence among hospitalized patients: A study using smart clinical data warehouse
Source: PLoS One. 2025 Jan 8;20(1):e0316068. doi: 10.1371/journal.pone.0316068 (PMC11709256; doi:10.1371/journal.pone.0316068)
Supplement: S1 Table — (DOCX) [file pone.0316068.s001.docx]

S1 Table. Baseline characteristics between overall stroke, ischemic and hemorrhagic stroke.

|  | Stroke (+)  (n=1853) | Ischemic stroke  (n=783) | Hemorrhagic stroke  (n=1070) | p-value |
| --- | --- | --- | --- | --- |
| Age | 66.8 (14.9) | 65.0 (15.1) | 68.2 (14.6) | <0.001 |
| Male | 998 (53.9) | 436 (55.7) | 562 (52.5) | 0.32 |
| HTN | 930 (50.2) | 358 (45.7) | 572 (53.5) | 0.004 |
| DM | 525 (28.3) | 191 (24.4) | 334 (31.2) | <0.001 |
| CAD | 183 (9.9) | 71 (9.1) | 112 (10.5) | 0.33 |
| prior stroke | 189 (10.2) | 79 (10.1) | 110 (10.3) | 0.43 |
| Prior malignancy | 356 (19.2) | 155 (19.8) | 201 (18.8) | <0.001 |
| Hyperlipidemia | 176 (9.5) | 74 (9.5) | 102 (9.5) | 0.45 |
| AF | 252 (13.6) | 195 (24.9) | 57 (5.3) | <0.001 |
| Alcohol | 309 (16.7) | 162 (20.7) | 147 (13.7) | <0.001 |
| Smoking | 234 (12.6) | 114 (14.6) | 120 (11.2) | <0.001 |
| Prior antithrombotics | 263 (14.2) | 108 (13.8) | 155 (14.5) | 0.92 |
| MEWS |  | 4 (3-5) | 6 (4-6) | <0.001 |
| 0-4 |  | 573 (73.2) | 565 (52.8) |  |
| >4 | 1138 (61.4) | 210 (26.8) | 505 (47.2) |  |
| NEWS | 715 (38.6) | 4 (3-6) | 6 (4-8) | <0.001 |
| 0-4 |  | 434 (55.4) | 346 (32.3) |  |
| >4 | 783 (42.3) | 349 (44.6) | 724 (67.7) |  |
