# Supplementary material for: Comparison of early warning scoring systems for predicting stroke occurrence among hospitalized patients: A study using smart clinical data warehouse
Source: PLoS One. 2025 Jan 8;20(1):e0316068. doi: 10.1371/journal.pone.0316068 (PMC11709256; doi:10.1371/journal.pone.0316068)
Supplement: S2 Table — (DOCX) [file pone.0316068.s002.docx]

S2 Table. Multivariate analysis showing MEWS and NEWS >4 on stroke occurrence.

|  | OR | 95%CI | p-value |  | OR | 95%CI | p-value |
| --- | --- | --- | --- | --- | --- | --- | --- |
| Age | 1.004 | 0.999-1.01 | 0.10 | Age | 1.002 | 0.998-1.01 | 0.31 |
| Male | 1.11 | 0.97-1.27 | 0.13 | Male | 1.16 | 1.02-1.33 | 0.03 |
| DM | 1.08 | 0.93-1.25 | 0.34 | DM | 1.05 | 0.90-1.21 | 0.55 |
| Prior malignancy | 1.49 | 1.25-1.77 | <0.001 | Prior malignancy | 1.39 | 1.17-1.65 | <0.001 |
| AF | 4.82 | 3.79-6.14 | <0.001 | AF | 4.68 | 3.66-5.98 | <0.001 |
| alcohol | 0.86 | 0.71-1.03 | 0.11 | alcohol | 0.86 | 0.71-1.03 | 0.10 |
| smoking | 0.87 | 0.71-1.07 | 0.18 | smoking | 0.91 | 0.74-1.12 | 0.36 |
| MEWS>4 | 13.90 | 11.51-16.79 | <0.001 | NEWS>4 | 7.25 | 6.35-8.27 | <0.001 |
