# Supplementary material for: Comparison of early warning scoring systems for predicting stroke occurrence among hospitalized patients: A study using smart clinical data warehouse
Source: PLoS One. 2025 Jan 8;20(1):e0316068. doi: 10.1371/journal.pone.0316068 (PMC11709256; doi:10.1371/journal.pone.0316068)
Supplement: S3 Table — (DOCX) [file pone.0316068.s003.docx]

S3 Table. Multivariate analysis showing MEWS and NEWS >4 on ischemic and hemorrhagic stroke occurrence.

|  | Ischemic stroke | | | | |  | Hemorrhagic stroke | | | | |
| --- | --- | --- | --- | --- | --- | --- | --- | --- | --- | --- | --- |
|  | OR | 95%CI |  | OR | 95%CI |  | OR | 95%CI |  | OR | 95%CI |
| Age | 0.996 | 0.99-1.002 | Age | 0.995 | 0.99-1.001 | Age | 1.01 | 1.00-1.02 | Age | 1.01 | 1.00-1.01 |
| Male | 1.06 | 0.90-1.26 | Male | 1.08 | 0.91-1.28 | Male | 1.10 | 0.94-1.28 | Male | 1.15 | 0.99-1.35 |
| HTN | 0.92 | 0.77-1.10 | HTN | 0.93 | 0.78-1.11 | HTN | 0.998 | 0.85-1.18 | HTN | 1.02 | 0.87-1.20 |
| DM | 0.96 | 0.79-1.17 | DM | 0.95 | 0.78-1.16 | DM | 1.16 | 0.98-1.38 | DM | 1.12 | 0.94-1.32 |
| Prior malignancy | 1.47 | 1.19-1.80 | Prior malignancy | 1.44 | 1.17-1.76 | Prior malignancy | 1.19 | 0.98-1.45 | Prior malignancy | 1.12 | 0.93-1.37 |
| AF | 8.11 | 6.47-10.15 | AF | 7.98 | 6.37-10.00 | AF | 0.47 | 0.34-0.66 | AF | 0.48 | 0.35-0.66 |
| alcohol | 1.07 | 0.85-1.34 | alcohol | 1.06 | 0.85-1.33 | alcohol | 0.74 | 0.59-0.94 | alcohol | 0.75 | 0.60-0.94 |
| Smoking | 0.94 | 0.73-1.22 | Smoking | 0.97 | 0.75-1.24 | Smoking | 0.86 | 0.67-1.10 | Smoking | 0.90 | 0.70-1.15 |
| MEWS>4 | 2.15 | 1.78-2.60 | NEWS>4 | 2.00 | 1.70-2.36 | MEWS>4 | 9.75 | 8.27-11.50 | NEWS>4 | 7.80 | 6.72-9.07 |
