# Supplementary material for: Comparison of early warning scoring systems for predicting stroke occurrence among hospitalized patients: A study using smart clinical data warehouse
Source: PLoS One. 2025 Jan 8;20(1):e0316068. doi: 10.1371/journal.pone.0316068 (PMC11709256; doi:10.1371/journal.pone.0316068)
Supplement: S4 Table — (DOCX) [file pone.0316068.s004.docx]

S4 Table. Multivariate analysis showing MEWS and NEWS on stroke occurrence using PSM cohort.

|  | OR | 95%CI | p-value |  | OR | 95%CI | p-value |
| --- | --- | --- | --- | --- | --- | --- | --- |
| Age | 0.996 | 0.99-1.00 | 0.12 | Age | 0.99 | 0.99-0.999 | 0.03 |
| Male | 1.06 | 0.92-1.24 | 0.42 | Male | 1.10 | 0.95-1.28 | 0.21 |
| HTN | 1.04 | 0.89-1.22 | 0.61 | HTN | 1.06 | 0.91-1.24 | 0.47 |
| DM | 0.87 | 0.74-1.02 | 0.10 | DM | 0.85 | 0.72-0.998 | 0.047 |
| Prior malignancy | 0.82 | 0.68-0.98 | 0.03 | Prior malignancy | 0.77 | 0.64-0.92 | 0.004 |
| AF | 2.30 | 1.80-2.92 | <0.001 | AF | 2.24 | 1.75-2.86 | <0.001 |
| alcohol | 1.07 | 0.86-1.33 | 0.57 | alcohol | 1.03 | 0.83-1.29 | 0.77 |
| smoking | 1.06 | 0.83-1.35 | 0.63 | smoking | 1.13 | 0.89-1.44 | 0.32 |
| MEWS>4 | 12.5 | 9.92-15.75 | <0.001 | NEWS>4 | 6.71 | 5.75-7.83 | <0.001 |
